# Acupuncture for Chronic Urinary Retention due to Spinal Cord Injury: A Systematic Review

**DOI:** 10.1155/2016/9245186

**Published:** 2016-04-13

**Authors:** Jia Wang, Yanbing Zhai, Jiani Wu, Shitong Zhao, Jing Zhou, Zhishun Liu

**Affiliations:** ^1^Department of Acupuncture, Guang'anmen Hospital, China Academy of Chinese Medical Sciences, No. 5 Beixiange Street, Xicheng District, Beijing 100053, China; ^2^Beijing University of Chinese Medicine, No. 11 North Third Ring Road, Chaoyang District, Beijing 100029, China

## Abstract

No systematic review has been published on the use of acupuncture for the treatment of chronic urinary retention (CUR) due to spinal cord injury (SCI). The aim of this review was to assess the effectiveness and safety of acupuncture for CUR due to SCI. Three randomized controlled trials (RCTs) including 334 patients with CUR due to SCI were included. Meta-analysis showed that acupuncture plus rehabilitation training was much better than rehabilitation training alone in decreasing postvoid residual (PVR) urine volume (MD −109.44, 95% CI −156.53 to −62.35). Likewise, a combination of acupuncture and aseptic intermittent catheterization was better than aseptic intermittent catheterization alone in improving response rates (RR 1.23, 95% CI 1.10 to 1.38). No severe adverse events were reported. In conclusion, acupuncture as a complementary therapy may have a potential effect in CUR due to SCI in decreasing PVR and improving bladder voiding. Additionally, acupuncture may be safe in treating CUR caused by SCI. However, due to the lack of high quality RCTs, we could not draw any definitive conclusions. More well-designed RCTs are needed to provide strong evidence.

## 1. Introduction

Urinary retention is described as a bladder that either empties incompletely or does not empty at all [1]. The International Continence Society (ICS) defines chronic urinary retention (CUR) as “a non-painful bladder, which remains palpable or percussable [*sic*] after the patient has passed urine” [2]. Most studies and the UK National Institute for Health and Clinical Excellence guidelines for lower urinary tract symptoms describe CUR as either a postvoid residual (PVR) urine volume > 300 mL in patients who are able to void or PVR urine volume > 1,000 mL in patients who are unable to void [3–8]. CUR can be caused by obstructive, neurologic, myogenic, or other pathogeneses, such as spinal cord injury (SCI), pelvic nerve injury, peripheral neuropathy, and detrusor overdistention injury [9, 10]. SCI is an important nonobstructive pathogenesis of CUR, which can disrupt the reflex circuitry controlling micturition [11]. To the best of our knowledge, the exact incidence and prevalence of CUR due to SCI are still unclear. This disease has a serious impact on patients' health and their quality of life. Long-term neglect of CUR may lead to chronic urinary tract infections, upper urinary tract damage, and renal failure [1, 12, 13]. Therefore, timely diagnosis and treatment are vital.

CUR due to SCI can be treated by pelvic floor training [2, 14], sacral neuromodulation (SNM) [15, 16], and intravesical electrostimulation (IVES) [17, 18]. However, IVES provides only short-term efficacy, and the potential complications of SNM include implant infection, pain, and superficial dehiscence [15, 17, 18]. CUR due to SCI can also be relieved using an intermittent or indwelling catheter [4, 19, 20]; although these relief methods have some benefits, catheterization cannot help patients restore their voiding function. Furthermore, long-term catheterization may be associated with complications such as discomfort, urethral injury, and urinary tract infection [19–24].

Acupuncture originates from ancient China and has been used to manage various clinical disorders for thousands of years. Although acupuncture plays an important role in Traditional Chinese Medicine (TCM) and is commonly used for treating CUR due to SCI in Mainland China, no systematic review of this treatment for CUR due to SCI exists; hence, the effectiveness and safety of this treatment remain unclear. The aim of our study was to assess the effectiveness and safety of acupuncture for CUR due to SCI.

## 2. Methods and Analysis

### 2.1. Search Strategy

The search strategy was decided according to the guidance of the* Cochrane Handbook*. The key words included “urinary retention”, “chronic urinary retention”, “CUR”, “chronic retention of urine”, “spinal cord injury”, “traumatic myelopathy”, “spinal cord laceration”, “post traumatic myelopathy”, “spinal cord contusion”, “spinal cord trauma”, “acupuncture”, “manual acupuncture”, “electrical acupuncture”, “auricular acupuncture”, “scalp needle”, and “elongated needle”. We electronically searched the following databases from their inception: PubMed, EMBASE, Cochrane Central Register of Controlled Trials, ClinicalTrials.gov, China National Knowledge Infrastructure (CNKI), Wan-Fang Database, Chinese Scientific Journal Database (VIP database), and the Chinese Biomedical Literature Database (CBM). In addition, CNKI was searched for unpublished articles, including conference articles and Chinese Doctoral and Master's theses.

### 2.2. Criteria for Study Inclusion in This Review

#### 2.2.1. Type of Studies

Only randomized controlled trials (RCTs) investigating acupuncture for CUR due to SCI in English or Chinese without restrictions on publication status were included. Nonrandomized studies, quasi-randomized studies, retrospective studies, case reports, case series, literature reviews, animal experiments, clinical empirical summaries, journal indexes, and epidemiological surveys were excluded.

#### 2.2.2. Type of Participants

Patients who had experienced CUR due to SCI, which is defined as a nonpainful bladder that remained palpable or percussible after they had passed urine, were included. Additionally, patients who are able to void with a PVR > 300 mL or unable to void with a PVR > 1,000 mL were included. There were no limitations on sex, age, and race. Patients with CUR caused by other pathogeneses, such as prostate cancer, bladder tumors, and benign prostatic hyperplasia, were excluded [25].

#### 2.2.3. Type of Intervention

All types of TCM acupuncture, including manual acupuncture, electrical acupuncture, auricular acupuncture, elongated needle, and scalp needle, were included. Moxibustion, warm needling, fire needling, acupoint injection, and other non-TCM acupuncture therapies were excluded. RCTs that had control groups with no intervention, sham acupuncture, placebo control, drug therapy, rehabilitation training (such as bladder training), or other conservative treatments such as catheterization were eligible. RCTs involving acupuncture combined with another therapy were also included if that other therapy was the same in both experimental and control groups. Trials comparing different types of acupuncture and acupoints were excluded. Acupuncture plus Chinese medicine compared with Chinese medicine was excluded, and acupuncture plus moxibustion compared with moxibustion was also excluded.

#### 2.2.4. Type of Outcomes

Each trial was required to include at least one of the following outcomes: ① the change of PVR from baseline after treatment and follow-up; ② response rates (proportion of patients improved): effective (regaining the ability to automatically urinate with PVR < 100 mL) and ineffective (regaining partial ability to automatically urinate with PVR ≥ 100 mL or keeping the inability to automatically urinate after treatment); ③ health-related quality of life (HRQL) measured by the Short-Form Health Survey Questionnaire (SF-36) or other internationally accepted scoring scales; ④ adverse events during the scheduled treatment time and follow-up. Any available information about safety was extracted from all clinical studies of acupuncture for CUR due to SCI, including other non-RCTs.

### 2.3. Study Selection

**Figure 1:**
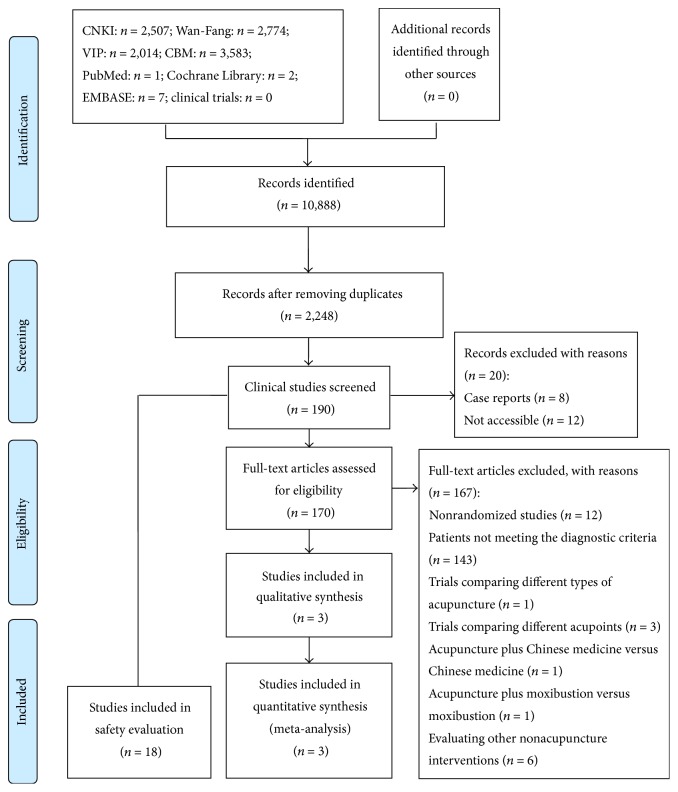
Flow diagram for the process of selecting eligible RCTs.

Two reviewers (Jia Wang and Yanbing Zhai) independently screened titles and abstracts of obtained trials to select articles for full-text assessment and then independently screened full-text papers to confirm eligibility of the trials. We included studies that met the predetermined inclusion criteria listed above. Any disagreements were resolved by consensus discussion between reviewers or with a third party (Zhishun Liu) if necessary. A PRISMA flow diagram was used to describe the process of study selection (Figure 1).

### 2.4. Data Extraction and Management

Two reviewers (Jia Wang and Yanbing Zhai) independently used a predesigned data extraction form for rigorous data collection, including general information such as authors, year of publication, study size, gender and age of the participants, treatment process, and details of the control, as well as trial characteristics such as randomization, allocation concealment, blinding, incomplete outcome data, selective reporting, and other outcomes.

### 2.5. Assessment of Risk of Bias in Included Studies

Two authors (Jia Wang and Yanbing Zhai) independently evaluated the methodological quality using the* Cochrane Handbook for Systematic Review of Interventions*, Version 5.1.0 [26], which comprised seven domains: random sequence generation (selection bias), allocation concealment (selection bias), blinding of participants and personnel (performance bias), blinding of outcome assessment (detection bias), incomplete outcome data (attrition bias), selective reporting (reporting bias), and other bias. Each domain was categorized into three levels: low risk, unclear risk, and high risk.

### 2.6. Measures of Treatment Effect

For dichotomous outcomes, the combination of the risk ratio (RR) and 95% confidence intervals (CI) was applied. For continuous outcomes, the combination of the mean difference (MD) and 95% CI was used.

### 2.7. Dealing with Missing Data

We tried to contact the first or corresponding authors by telephone or email to obtain the missing information. In the case of unobtainable missing data, we used the intention-to-treat (ITT) analysis for dichotomous outcomes if possible.

### 2.8. Assessment of Heterogeneity

The Higgins *I* ^2^ test was applied for heterogeneity prior to the meta-analysis to find out if the included studies were heterogeneous. If the *I* ^2^ statistics were less than 50%, the heterogeneity was deemed acceptable. If the *I* ^2^ statistics were greater than 50%, this indicated significant heterogeneity among the studies.

### 2.9. Assessment of Reporting Bias

Funnel plots were used to assess the reporting bias if there were more than 10 studies included in one meta-analysis. The plots were assessed visually or by Egger's test [27].

### 2.10. Data Synthesis

RevMan V.5.3.1 software from the Cochrane Collaboration was used for the data synthesis [26]. If there was no significant heterogeneity, a combination of RR and 95% CI of each study using the fixed-effect model was applied for dichotomous data, and a combination of MD and 95% CI of each study using the fixed-effect model was applied for continuous data. If significant heterogeneity was detected, the random-effect model was used. For all the analyses, a *P* value of less than 0.05 was considered statistically significant. For data that were not appropriate for undergoing quantitative synthesis, we performed a narrative review of the evidence.

### 2.11. Subgroup Analysis and Sensitivity Analysis

Subgroup analysis was performed according to the use of sham/placebo or positive control when there were more than three trials in each group. A sensitivity analysis was conducted to assess the potential bias of individual trials on the outcome of the meta-analysis for this review if possible.

## 3. Results

### 3.1. Study Selection

Through electronic searching, 10,888 records were identified. 2,248 records were eligible for screening after duplicate records were removed. After screening the title and abstract, 190 clinical studies were remained. Then, approximately 170 studies potentially met the inclusion criteria, and these studies were identified for full-text screening. Ultimately, only three RCTs were included for evaluating the effectiveness of acupuncture treatment, while 18 trials were included for safety evaluation [28–30]. All these trials were conducted in China and published in Chinese. The process of study selection was shown in Figure 1.

### 3.2. Characteristics of Included Trials

The characteristics of included trials were summarized in Table 1.

#### 3.2.1. Patients

A total of 334 participants in three trials were included, with sample sizes ranging from 70 to 132. The age of patients ranged from 11 to 71 years. The disease course ranged from one month to three years. Spinal cord injury was the etiology in all the included trials [28–30].

#### 3.2.2. Acupuncture Interventions

Electroacupuncture was applied in two trials [29, 30], and manual acupuncture was used in one trial [28]. In this trial, manual acupuncture performed was based on disease diagnosis but not based on syndrome differentiation [28]. Patients received acupuncture therapy once a day, and each treatment lasted for 20 [29, 30] or 30 [28] minutes. The treatment duration ranged from two weeks [29, 30] to eight weeks [28]. Zhongji (CV3) [28–30], Qihai (CV6) [28–30], and Guanyuan (CV4) [28–30] were the most frequently used points, with an incidence of 100.0% among these three RCTs. Pangguangshu (BL28) [30], Shenshu (BL23) [29, 30], Qugu (CV2) [29, 30], Yinlingquan (SP9) [29, 30], and Sanyinjiao (SP6) [29, 30] were the next most commonly used acupoints, with an incidence of 66.7% among these three RCTs. Other acupoints mentioned in the included trials were Yaoyangguan (GV3) [28], Mingmen (GV4) [28], Ciliao (BL32) [30], Shangliao (BL31) [30], Zhongliao (BL33) [30], and Xialiao (BL34) [30]. All these acupoints selected were based on disease diagnosis but not due to nerves [28–30]. While acupuncturing, De Qi was obtained of all these acupoints.

#### 3.2.3. Control Interventions

One trial compared acupuncture plus rehabilitation training with rehabilitation training [28]. In this trial, rehabilitation training included bladder sphincter training and urination training. Two trials compared acupuncture plus aseptic intermittent catheterization with aseptic intermittent catheterization [29, 30]. In these three trials, some supportive therapies were applied in both experimental and control groups, including water quantity control [29, 30], neurotrophic drugs [28], dehydrant agents [28], and regulating water, electrolyte, and acid-base balance drugs [28].

#### 3.2.4. Outcome Measures

One trial evaluated postvoid residual (PVR) urine volume [28], while the other two trials evaluated response rates [29, 30]. No trial measured health-related quality of life (HRQL).

### 3.3. Risk of Bias in the Included RCTs

All three included trials mentioned randomization through the use of random number tables [28–30]. Two trials performed allocation concealment using opaque envelopes [28, 30], and one trial did not perform allocation concealment [28]. Because of the nature of acupuncture, none of the included trials applied the method of blinding acupuncturists and participants. One trial applied the method of blinding outcome assessment [28], but the other two trials did not apply it [29, 30]. One trial reported relevant information regarding drop-outs because of the change in the condition of diseases, but there was no information about the principle used for dealing with the missing data [28]. There was also no information relating to intention-to-treat (ITT) analysis. Therefore, all these trials were evaluated as having high risk of bias. The details of the risks of bias of each trial were presented in Table 2.

### 3.4. Effects of Interventions

Three trials were divided into two parts to conduct meta-analysis according to different types of comparison groups. Then, trials with similarities were pooled together. Subgroup analysis was not conducted, as there were an insufficient number of studies included in this review.

#### 3.4.1. Acupuncture plus Aseptic Intermittent Catheterization versus Aseptic Intermittent Catheterization

*Postvoid Residual (PVR) Urine Volume*. In this comparison, no trials applied PVR as the outcome measurement.

**Figure 2:**
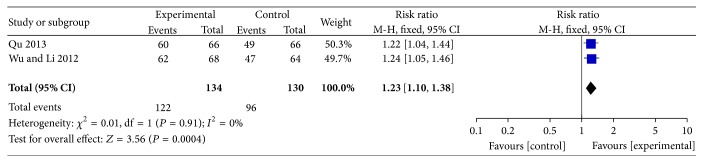
Forest plot of the effect of acupuncture plus aseptic intermittent catheterization versus aseptic intermittent catheterization on response rates using the fixed model.

*Response Rates.* Two trials reported response rates, and the RR was 1.23 (95% CI 1.10 to 1.38) using the fixed model [29, 30], which showed that there was a significant difference in the increase in response rates upon comparing acupuncture plus aseptic intermittent catheterization with aseptic intermittent catheterization alone (Figure 2).

*Health-Related Quality of Life (HRQL)*. These two trials did not report the outcome of the improvement of life quality during treatment course and follow-up [29, 30].

#### 3.4.2. Acupuncture plus Rehabilitation Training versus Rehabilitation Training

**Figure 3:**
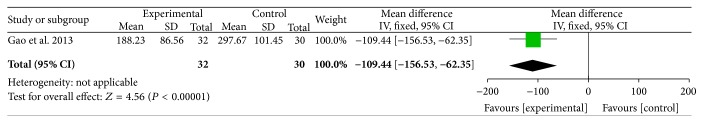
Forest plot of the effect of acupuncture plus rehabilitation training versus rehabilitation training on PVR using the fixed model. PVR: postvoid residual.

*PVR*. The MD was −109.44 (95% CI −156.53 to −62.35) using the fixed model in one trial [28], which showed that acupuncture plus rehabilitation training was much better than rehabilitation training alone in decreasing PVR from baseline (Figure 3). 

*Response Rates and HRQL*. This trial did not mention any information about the change of response rates and HRQL for CUR patients caused by SCI [28].

### 3.5. Safety

There were 18 clinical trials eligible to evaluate the safety of acupuncture for CUR due to SCI. In these trials, 921 patients were treated by acupuncture or by acupuncture combined with other therapies. None of these 18 studies reported any severe adverse events related to acupuncture. And only one trial reported adverse events [31], which was a prospective cohort study of electrical acupuncture for CUR due to SCI in 153 patients. It reported the adverse events of pricking, faint, stuck needle, hematoma around the site of needling, infection, and pelvic floor dysfunction, which disappeared after the treatment withdrawal. However, it did not report the incidence of adverse events.

### 3.6. Publication Bias

As the number of included trials was <10, funnel plots were not applied to detect potential publication bias.

## 4. Discussion

Chronic urinary retention (CUR) is a common urological problem that greatly impacts patients' general health, quality of daily life [32], psychological states [32], and social contact. Prestudies reveal that acupuncture may have promising therapeutic effectiveness in urinary retention [31, 33]. This review had evaluated the effectiveness of acupuncture for CUR due to spinal cord injury (SCI). However, only three RCTs met the inclusion criteria.

### 4.1. Summary of Effectiveness

In this review, the main findings of effectiveness were the notion that acupuncture plus rehabilitation training was much better than rehabilitation training alone in decreasing postvoid residual (PVR) urine volume for CUR due to SCI [28] and a combination of acupuncture and aseptic intermittent catheterization was more effective than aseptic intermittent catheterization alone in improving response rates for CUR due to SCI [29, 30].

PVR measurement is an important component in the assessment of voiding dysfunction, including CUR due to SCI [34–36]. Accurate determination of PVR is of significant importance for the diagnosis and treatment of urinary retention [36, 37]. It is also a valuable parameter for evaluating the effectiveness of treatments [36]. The decrease in PVR after treatments can reveal the improvement of voiding function. However, in this review the evidence regarding PVR is limited, as only one RCT with a small sample size performed this measurement [28]. Moreover, this trial did not assess patients during the follow-up period [28], and the information about the drop-outs and other missing data was also inadequate, which may have led to attrition bias. Therefore, the evidence on the effectiveness of acupuncture in decreasing PVR was insufficient. Future studies should pay more attention to this outcome measure and provide stronger evidence.

The improvement of response rates can also reflect the effectiveness of acupuncture for CUR due to SCI. In this review, two trials evaluated response rates [29, 30], which were divided into three grades: cured (restoration of the ability to automatically urinate, disappearance of urinary retention symptoms, and PVR < 80 mL); effective (restoration of the ability to automatically urinate, disappearance of urinary retention symptoms, and 80 mL < PVR < 100 mL), and ineffective (persistence of the inability to automatically urinate, PVR > 100 mL). In clinical practice, PVR < 30 mL can be regarded as insignificant [38]. Meanwhile, PVR < 100 mL for CUR patients due to SCI may indicate the complete or partial restoration of lower urinary tract function and the reduction of upper urinary tract impairment [39]. In this review, we merged the cured and effective components into a single effective component.

In these two trials, the information related to the exact process of randomization and allocation concealment was too scarce [29, 30], which may have led to high risks of selection bias. Additionally, these two trials did not blind the outcome assessment [29, 30], which may have caused detection bias. In addition, all these trials were conducted in China and published in Chinese [29, 30], which may have led to publication bias. The exact process of this measurement and the exact data of patients' symptoms and PVR were also not provided, which may have led to some other potential bias. Therefore, the evidence of acupuncture for CUR due to SCI in improving response rates was still inadequate.

In addition, CUR due to SCI can greatly impact patients' health-related quality of life (HRQL) [32] and the improvement of HRQL can also be an important outcome for the effectiveness. Moreover, SF-36 and other internationally accepted scoring scales can help evaluate the patients' general health, which can be applied to measure the HRQL. However, none of the included trials mentioned any information about HRQL. Thus, the evidence on the effectiveness of acupuncture in improving HRQL was still unobtainable in this review. Therefore, we suggested that future studies should attach more importance to the outcome of HRQL.

Based on this evidence, acupuncture may have a potential therapeutic effect in decreasing PVR and improving response rates for CUR due to SCI. However, the evidence was still too weak to draw any firm conclusions.

### 4.2. Summary of Safety

Acupuncture therapy may also be related to some adverse events such as pricking, redness, ecchymosis [40], hematoma [31], fainting, and bleeding in the local skin [41]. Though these adverse events are minor, the safety management of acupuncture should also be taken into account. In this systematic review, 18 clinical trials were eligible to evaluate the safety of acupuncture for CUR due to SCI.

Adverse events related to acupuncture occurred in only one trial, including pricking, fainting, stuck needles, hematoma around the site of needling, infection, and pelvic floor dysfunction, which were all minor. The other 17 clinical trials did not report any adverse events. No severe adverse events were reported, which indicated that acupuncture was a safe treatment for CUR due to SCI. However, as no trials mentioned any relevant information concerning the follow-up, the long-term safety of acupuncture still remains uncertain. Therefore, we suggest that the long-term safety of acupuncture should also be taken into account in future research.

### 4.3. Limitations of This Review

There were some limitations in our systematic review. First, though we attempted to search CNKI for conference articles and Doctoral and Master's theses, it was difficult to obtain all the unpublished articles. Moreover, because of the language barrier, we only searched databases in Chinese and English; Korean, Japanese, and other foreign databases were not covered. Thus, some relevant clinical trials may have been missed. Second, the three included trials were all conducted in China and most of them omitted the exact process of randomization and allocation concealment, which would lead to selection bias. None of the trials blinded the participants and acupuncturists to the treatment, which may have elicited performance bias; two trials did not blind the outcome assessment, which would lead to detection bias; one trial had incomplete outcome data, which would yield attrition bias. These high risks of bias may have influenced our final conclusions. Third, although we tried our best to contact the corresponding or first authors of each included RCT by phone or email, some missing information still remained unobtainable. Finally, the long-term effectiveness of acupuncture could not be determined as none of the included trials assessed the patients at follow-up.

### 4.4. Implications for Practice and Research

In this review, all the included trials were of small sample size and had high risk of bias. Though the data showed effectiveness of acupuncture in CUR due to SCI, the evidence was still insufficient. More attention should be paid to the outcome measures of PVR and HRQL. What is more, whether acupuncture had effect on CUR caused by other pathogeneses remained unknown. Future studies should attach more importance to other types of CUR. Moreover, the long-term effectiveness of acupuncture for CUR due to SCI remained unclear. Therefore, multicenter RCTs with large-scale and long-term follow-up should be conducted to provide more evidence.

## 5. Conclusions

Based on this systematic review, acupuncture may have some positive effectiveness in decreasing PVR and in improving response rates for chronic urinary retention (CUR) due to spinal cord injury (SCI). Additionally, acupuncture may be safe for treating CUR due to SCI. However, the evidence is still limited due to the lack of high quality trials. RCTs with large samples and high methodological quality are still needed in further clinical research. A convincing demonstration of the positive effectiveness and safety of acupuncture will have valuable implications for future clinical practice.

## Figures and Tables

**Table 1:** Characteristics of included studies.

Study ID	Sample size (T/C)	Age, y	Etiology	Disease course	Intervention	Control	Treatment duration	Follow-up	Outcomes
Gao et al. 2013 [28]	70	T: 37.20 ± 7.09	SCI	T: 48.34 ± 10.12 d	Acupuncture + RT, 30 min, Qd	RT	8 wk	No	PVR
	35/35	C: 35.20 ± 8.12		C: 46.03 ± 8.33 d					

Qu 2013 [29]	132	T: 8–66	SCI	T: 1 mon–3 y	EA + AIC 20 min, Qd	AIC	2 wk	No	RR
	66/66	C: 11–71		C: 2 mon–3 y					

Wu and Li 2012 [30]	132	T: 8–66	SCI	T: 13.6 ± 3.9 mon	EA + AIC 20 min, Qd	AIC	2 wk	No	RR
	68/64	C: 11–71		C: 14.3 ± 4.4 mon					

T = treatment group; C = control group; SCI = spinal cord injury; RT = rehabilitation training; EA = electrical acupuncture; AIC = aseptic intermittent catheterization; PVR = postvoid residual; RR = response rates.

**Table 2:** Methodological quality of included trials.

Study ID	A	B	C	D	E	F	G
Gao et al. 2013 [28]	−	−	+	−	+	?	?

Qu 2013 [29]	−	+	+	+	−	?	?

Wu and Li 2012 [30]	−	−	+	+	−	?	?

+ = high risk, ? = unclear, and − = low risk. A = random sequence generation (selection bias); B = allocation concealment (selection bias); C = blinding of participants and personnel (performance bias); D = blinding of outcome assessment (detection bias); E = incomplete outcome data (attrition bias); F = selective reporting (reporting bias); G = other bias.
